# Surgeon-General Newton L. Bates

**Published:** 1897-11

**Authors:** 


### Obituary.

---

Surgeon-general Newton L. Bates, United States Navy, died suddenly at the Shoreham Hotel, Washington, October 18, 1897, aged 59 years. Dr. Bates was an alumnus of Buffalo University Medical College, class of '61. Soon after graduation he entered the naval service as assistant surgeon and was attached to the naval hospital at New York. During the civil war he served on the "Seneca" with the South Atlantic squadron, in the naval laboratory at New York, and on the "Benton" with the Mississippi squadron. He was promoted to surgeon in 1865, was a member of the naval board of medical examiners from 1878 to 1880, and in 1881 was commissioned medical inspector. From 1880 to 1882 he was in charge of the naval hospital at Yokohama, and in 1888 he was advanced to the position of medical director. On October 1, 1897, he was appointed to succeed J. Rufus Tryon as surgeon-general and chief of the bureau of medicine and surgery in the navy department. He was compelled to take the oath of office in bed on account of illness, which was then supposed to be temporary in character.

Dr. Bates was an accomplished physician and a genial gentleman. His many amiable qualities attracted a large circle of friends and during his service he had been in every port in the civilised world, had met hundreds of distinguished people and was at graceful ease in the presence of everyone, whether a monarch or a private citizen. His loss will be deeply felt by his colleagues in the naval service, and especially will his demise come very near to the president of the United States, whose physician and friend he was.

